# Occlusion of the Right Ventricular Wall Branch of a Recessive Right Coronary Artery Resulting in Ventricular Fibrillation and Anterior ST-Segment Elevation—A Case Report

**DOI:** 10.3389/fcvm.2020.00124

**Published:** 2020-07-28

**Authors:** Harish Sharma, Sudhakar George

**Affiliations:** ^1^Institute of Cardiovascular Sciences, University of Birmingham, Birmingham, United Kingdom; ^2^University Hospitals Birmingham NHS Foundation Trust, Birmingham, United Kingdom

**Keywords:** RV branch occlusion, anterior ST elevation, ventricular fibrillation, myocardial infarction, right ventricular infarction

## Abstract

**Background:** Right ventricular (RV) infarction is as an extremely rare cause of isolated anterior ST-segment elevation. Occlusion of the RV branch in a recessive right coronary artery (RCA) causing isolated RV infarction and only anterior ST-elevation is extremely rare. To date, the handful of such cases reports do not describe any arrhythmia associated with this presentation. Although ventricular fibrillation (VF) has been well-documented with interruption of flow in the conus branch of the RCA, here we describe VF occurring in a patient with occlusion of the RV branch of a recessive RCA presenting with isolated anterior ST-segment elevation.

**Case:** A 51-year-old man presented with acute chest pain and isolated anterior ST-segment elevation on electrocardiogram (ECG). The patient developed ventricular fibrillation prior to coronary angiography requiring cardiopulmonary resuscitation. Coronary angiography revealed an unobstructed left coronary system and a recessive right coronary artery with ostial occlusion of the RV branch which was treated with a drug eluting balloon, resulting in resolution of the chest pain and ECG changes.

**Conclusion:** Isolated RV infarction due to RV branch occlusion can cause ECG changes mimic anterior left ventricular infarction. This presentation may be complicated by VF, even in the setting of a recessive RCA.

## Introduction

The ST-segment of an electrocardiogram (ECG) represents the time between ventricular depolarization and repolarization. In the majority of healthy individuals, the ST-segment is isoelectric. In the context of chest pain, ST-segment elevation in the left anterior precordial leads typically reflects anteroseptal infarction due to occlusion of the left anterior descending (LAD) artery. However, there are a broad range of differentials to consider (Table 1).

**Table 1:** Causes of electrocardiographic anterior ST-segment elevation.

**Causes of anterior ST-segment elevation on ECG**	
Coronary circulation	Anteroseptal infarction Spontaneous coronary artery dissection Prinzmetal angina Isolated right ventricular infarction
Inflammatory	Myocarditis Pericarditis
Cardiomyopathy	Takotsubo Cardiomyopathy
Metabolic	Hyperkalaemia Hypercalcaemia Hypothermia
Conduction abnormalities	Bundle branch block Brugada syndrome
Normal variants	Benign early repolarization

Right ventricular (RV) infarction is as an extremely rare cause of isolated anterior ST-segment elevation (1–4). RV infarction is caused by obstruction of flow either in the right coronary artery (RCA) proximal to the RV branch or in the RV branch itself. Proximal occlusion of a dominant RCA typically causes RV and inferior left ventricular (LV) wall infarction and results in ST-elevation in inferior and anterior leads, although the ST elevation in the inferior leads is often masked (due to anterior ST elevation leading to reciprocal inferior ST depression). Occlusion of the RV branch in a recessive RCA causing isolated RV infarction and only anterior ST-elevation is extremely rare. To date, the handful of such cases reports do not describe any arrhythmia associated with this presentation. Although ventricular arrhythmias have been well-documented with interruption of flow in the conus branch of the RCA, here we describe ventricular fibrillation occurring in a patient with occlusion of the RV branch of a recessive RCA presenting with isolated anterior ST-segment elevation.

## Case

**Figure 1:**
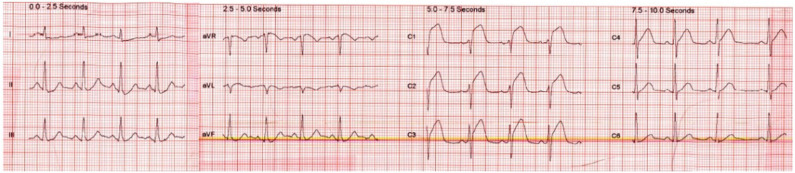
ECG on admission demonstrating anterior ST-segment elevation.

A 51-year-old man with a history of hypertension and cigarette smoking developed acute onset chest pain radiating to his jaw and back whilst at work. After the pain worsened over 2 h, the patient called for an ambulance and an electrocardiogram (ECG) revealed significant anterior ST elevation (see Figure 1).

**Figure 2:**
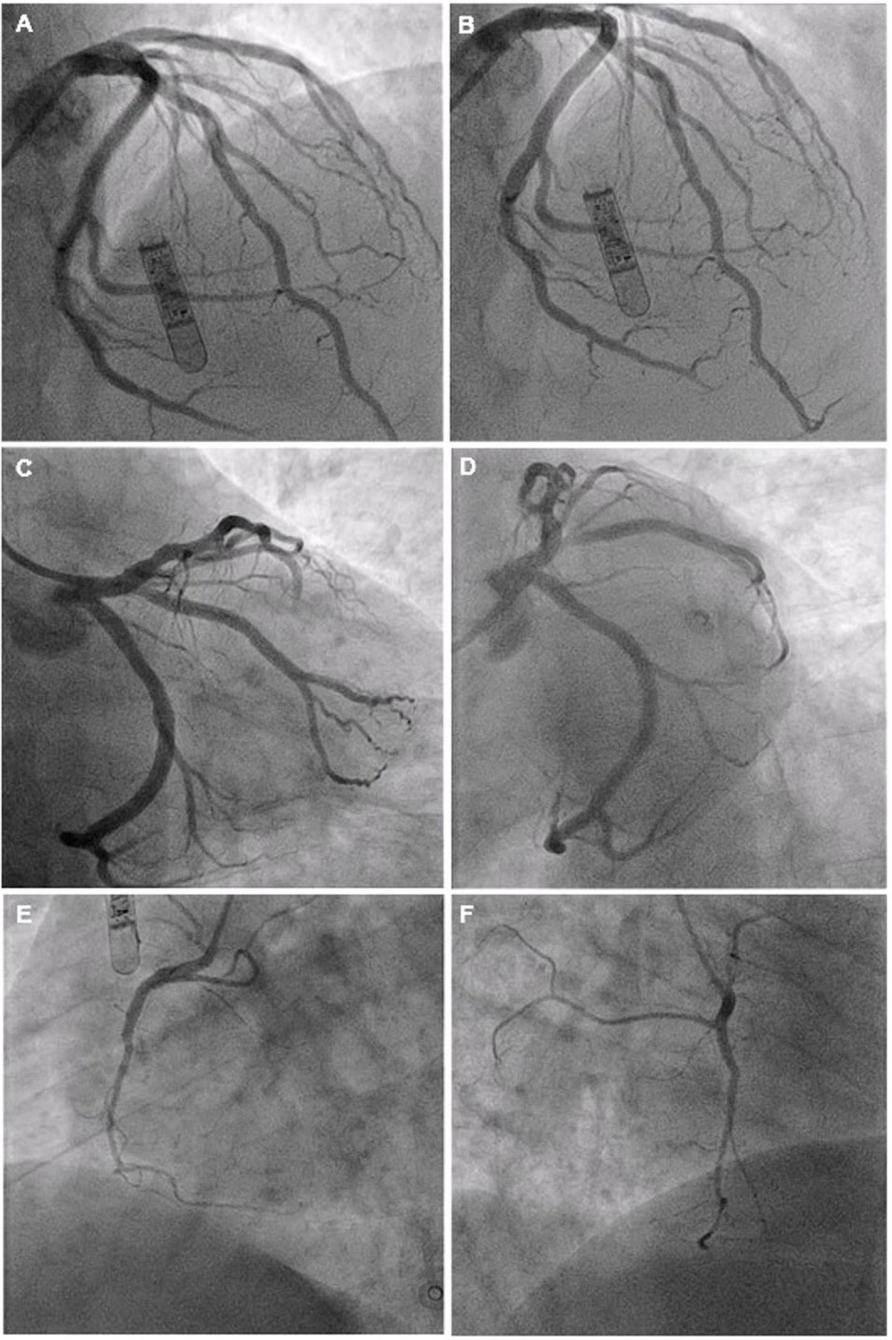
Diagnostic coronary angiography of left coronary artery system **(A–D)** and RCA **(E,F)** Views: **(A,B)** PA cranial; **(C)** PA caudal; **(D)** LAO Caudal; **(E)** LAO; **(F)** RAO.

The patient was treated as a suspected acute ST-elevation myocardial infarction (STEMI) and taken immediately to the cardiac catheterization laboratory. Before the procedure could commence, the patient developed ventricular fibrillation (VF) requiring 20 s of cardiopulmonary resuscitation and one direct current shock before return of spontaneous circulation. Coronary angiography via the right radial artery revealed a normal left main stem (LMS) and a dominant and unobstructed left circumflex artery (LCX). The left anterior descending artery (LAD) had good antegrade flow with only mild disease in the mid-vessel (Figures 2A–D, Video S1). Optical coherence tomography (OCT) examination of the vessel confirmed the absence of any acute plaque rupture in the proximal and mid LAD.

**Figure 3:**
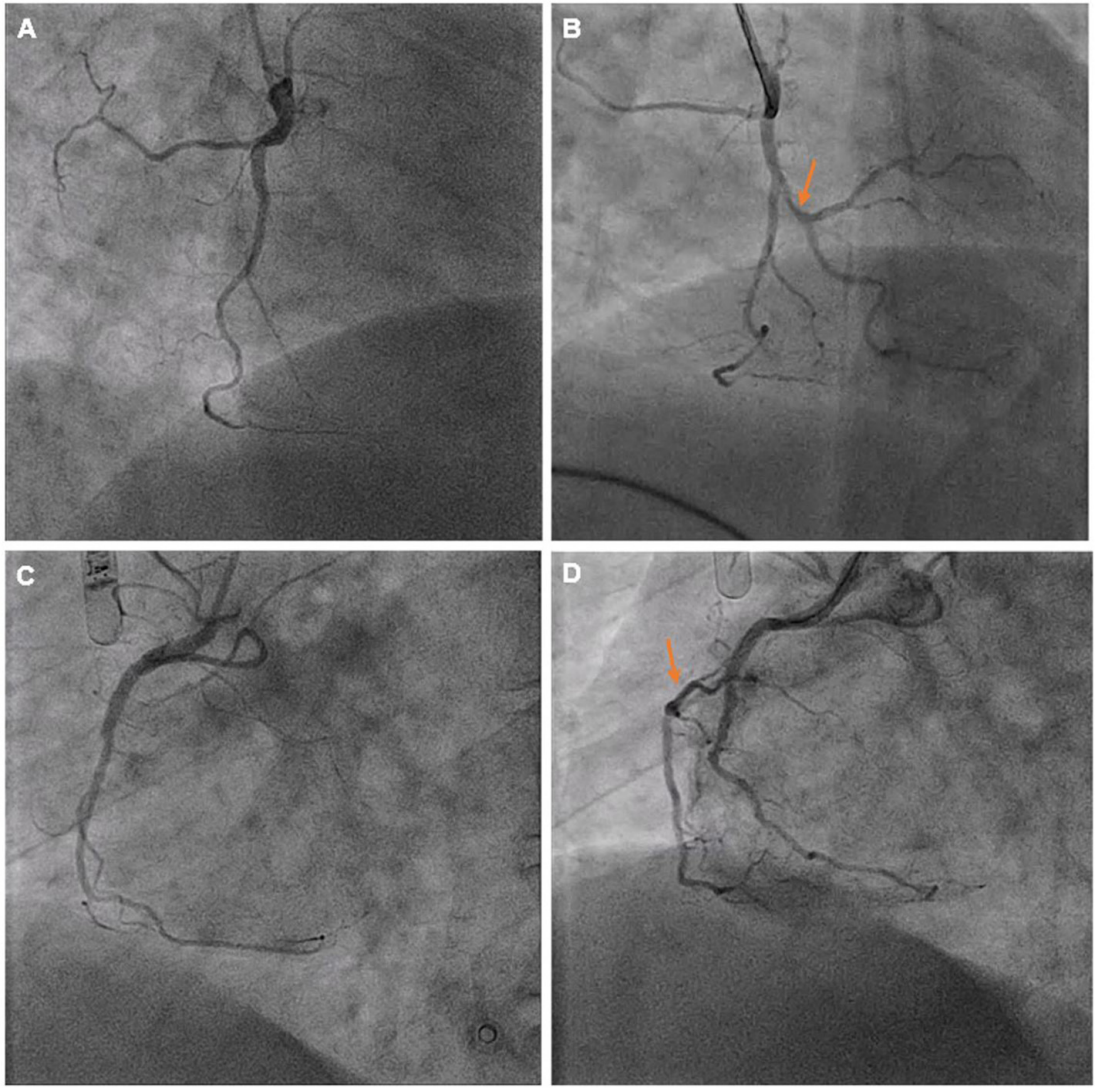
Views of the RCA before **(A,C)** and after **(B,D)** drug eluting balloon treatment. Markers indicate the RV branch which was occluded and subsequently recanalized following intervention.

Right coronary angiography revealed a recessive vessel with occlusion of the right ventricular (RV) branch at the ostium (Figures 2E,F, 3A,C). Flow was restored as a coronary wire was passed into the RV branch. The ostial lesion was treated with an IN.PACT Falcon 2.0 mm × 20 mm Falcon drug eluting balloon resulting in resolution of the chest pain and normalization of the ST segments (Figures 3B,D).

Following the procedure, the patient was transferred to the coronary care unit and was found to have a high-sensitivity troponin I of 22,880 ng/L. The following day, transthoracic echocardiography revealed normal left and right ventricular size and systolic function with no significant valvular disease. The patient experienced no further chest pain or arrhythmias and was subsequently discharged 2 days after admission.

## Discussion

RV infarction most commonly occurs due to occlusive coronary lesions in the RCA. It is associated with inferior ST-segment elevation due to concomitant infarction of the inferior left ventricular (LV) wall. Rarely, isolated RV infarction can occur, presenting with anterior ST-segment elevation. The ECG in such patients typically demonstrates significant ST-elevation in V1–2 and smaller ST-elevation in V5–6 (5).

RV infarction in such patients can be missed and treatment delayed as time is spent assessing the LAD, which more frequently causes anterior ST-segment elevation when flow is interrupted. This leads to significant clinical implications as patients with RV infarction are more at risk of hypotension requiring fluid resuscitation. Isolated RV infarction causing only anterior ST-segment elevation usually occurs in the setting of a recessive RCA. In this coronary pattern, the left circumflex artery supplies the inferior LV wall and the recessive RCA supplies only the RV free wall. Acute occlusion of the recessive RCA (or its RV branch), causes isolated RV infarction and the right ventricular electrical potentials are recorded in the left precordial leads producing anterior ST-segment elevation. The reason for this lies in the anatomical position of the RV which overlies the LV and thus infarction of the RV is recorded particularly in the first two precordial leads of the ECG (as seen in Figure 1).

While this presentation is rare, the particularly novel finding of this case is the occurrence of VF. Ventricular arrhythmias have been well-documented when the blood flow to the conus (first) branch of the RCA is interrupted (e.g., iatrogenic intubation of the conus branch by a coronary catheter during angiography and injection of contrast dye). The conus branch supplies the RVOT and ischaemia in this myocardial region can generate electrical disturbances and even VF. Such ventricular arrhythmias occurring due to occlusion of the RV (2nd) branch of a recessive RCA is extremely rare. Clinicians should be mindful that a patient presenting with isolated RV branch occlusion in a recessive RCA can present with only anterior ST-segment elevation and can develop life-threatening ventricular arrhythmias.

## Data Availability Statement

All datasets generated for this study are included in the article/Supplementary Material.

## Ethics Statement

Written informed consent was obtained from the individual (who is over the age of 16) for the publication of any potentially identifiable images or data included in this article.

## Author Contributions

Both authors contributed to the writing and proof-reading of the manuscript.

## Conflict of Interest

The authors declare that the research was conducted in the absence of any commercial or financial relationships that could be construed as a potential conflict of interest.

## Supplementary Material

Figure S1 Post-procedure ECG showing residual anterior ST-segment elevation.

Video S1 Left coronary angiogram (PA cranial view).

Video S2 Right coronary angiogram (RAO view) before intervention.

Video S3 Right coronary angiogram (RAO view) after intervention.
