# Supplementary figures and images for: Occlusion of the Right Ventricular Wall Branch of a Recessive Right Coronary Artery Resulting in Ventricular Fibrillation and Anterior ST-Segment Elevation—A Case Report
Source: Front Cardiovasc Med. 2020 Jul 28;7:124. doi: 10.3389/fcvm.2020.00124 (PMC7399019; doi:10.3389/fcvm.2020.00124)

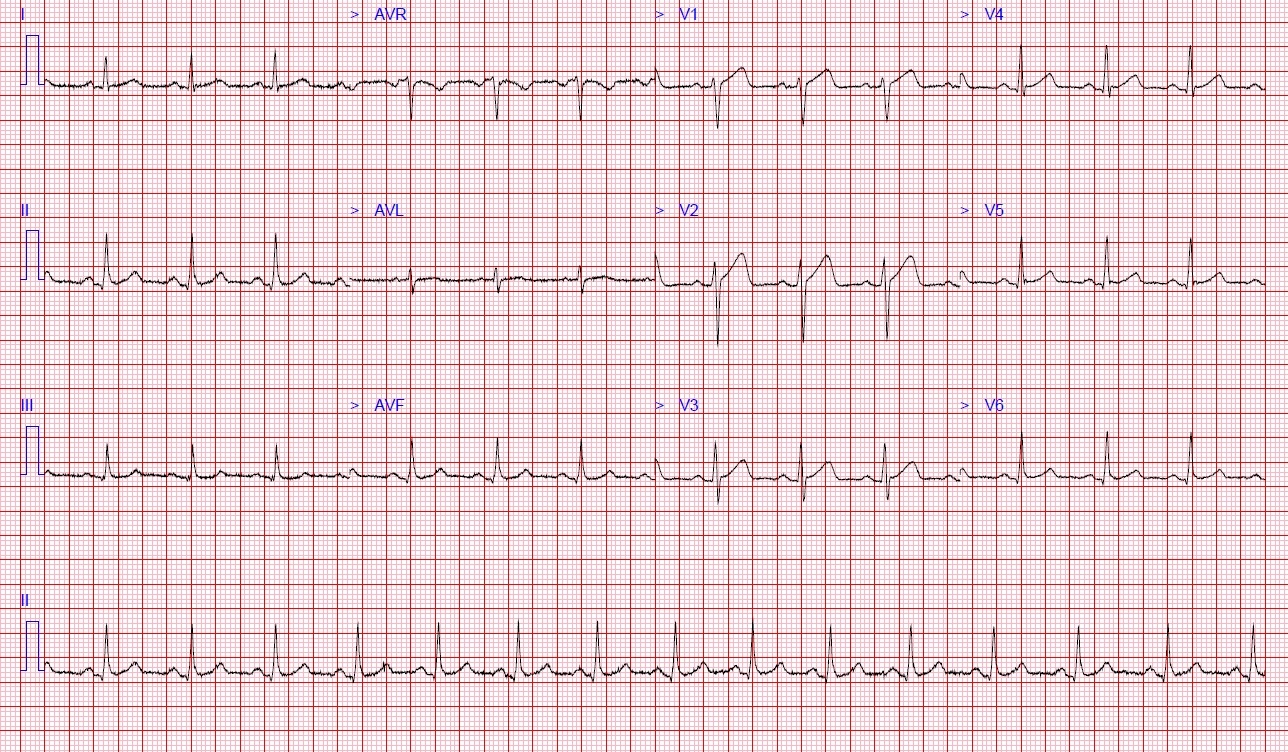

Supplement: Figure S1 — Post-procedure ECG showing residual anterior ST-segment elevation. [file Image_1.JPEG]
